# Thrombospondin-4 Is a Soluble Dermal Inflammatory Signal That Selectively Promotes Fibroblast Migration and Keratinocyte Proliferation for Skin Regeneration and Wound Healing

**DOI:** 10.3389/fcell.2021.745637

**Published:** 2021-09-23

**Authors:** Mariliis Klaas, Kristina Mäemets-Allas, Elizabeth Heinmäe, Heli Lagus, Claudia Griselda Cárdenas-León, Terje Arak, Mart Eller, Külli Kingo, Esko Kankuri, Viljar Jaks

**Affiliations:** ^1^Institute of Molecular and Cell Biology, University of Tartu, Tartu, Estonia; ^2^Department of Plastic Surgery and Wound Healing Centre, Helsinki University Hospital, University of Helsinki, Helsinki, Finland; ^3^Surgery Clinic, Tartu University Hospital, Tartu, Estonia; ^4^Dermatology Clinic, Tartu University Hospital, Tartu, Estonia; ^5^Department of Pharmacology, Faculty of Medicine, University of Helsinki, Helsinki, Finland

**Keywords:** skin regeneration, wound healing, burns, thrombospondin-4, fibroblasts, psoriasis

## Abstract

Thrombospondin-4 (THBS4) is a non-structural extracellular matrix molecule associated with tissue regeneration and a variety of pathological processes characterized by increased cell proliferation and migration. However, the mechanisms of how THBS4 regulates cell behavior as well as the pathways contributing to its effects have remained largely unexplored. In the present study we investigated the role of THBS4 in skin regeneration both *in vitro* and *in vivo*. We found that THBS4 expression was upregulated in the dermal compartment of healing skin wounds in humans as well as in mice. Application of recombinant THBS4 protein promoted cutaneous wound healing in mice and selectively stimulated migration of primary fibroblasts as well as proliferation of keratinocytes *in vitro*. By using a combined proteotranscriptomic pathway analysis approach we discovered that β-catenin acted as a hub for THBS4-dependent cell signaling and likely plays a key role in promoting its downstream effects. Our results suggest that THBS4 is an important contributor to wound healing and its incorporation into novel wound healing therapies may be a promising strategy for treatment of cutaneous wounds.

## Introduction

Cutaneous wound healing is a multifactorial process which involves both dermal and epidermal components that have important roles in restoring the full integrity of skin (Eming et al., 2014; Rousselle et al., 2019). The progressing steps of wound repair involve not only skin-resident cells but also circulating blood cells, controlled layers of cell-to-cell signaling and secretion of soluble factors and extracellular matrix (ECM) components that all play crucial roles in skin regeneration (Takeo et al., 2015; Rousselle et al., 2019). We have previously shown that changes in human skin regeneration patterns that cause alterations in the molecular composition of the recovered areas could be detected even within a year after healing of full thickness cutaneous wounds (Lagus et al., 2019). ECM proteins have a substantial role in this multi-step process, particularly in the proliferative phase of skin repair as they form a substrate for the migrating cells and support re-epithelialization (Rousselle et al., 2019). Matricellular proteins are ECM components that do not possess a structural role but regulate tissue homeostasis and wound healing. In line with this, several matricellular components such as thrombospondins, osteopontin, and tenascin are expressed at higher levels in healing wounds (Bornstein and Sage, 2002).

Thrombospondin-4 (THBS4) is a member of the thrombospondin family, which in turn belongs to the larger family of Ca-binding extracellular matrix proteins (Adams and Lawler, 2011). In general, thrombospondins are important regulators of multiple biological processes including cell-cell interactions (Adams, 2001), adhesion, embryonic development (O’Shea et al., 1990), synaptogenesis (Risher and Eroglu, 2012), tissue regeneration and remodeling (Adams and Lawler, 2011). THBS4 expression is normally very low in adult tissues but has been reported to massively increase after tissue damage and subsequent tissue repair and regeneration. Furthermore, high levels of THBS4 protein have been found in several pathological processes such as cancers (Singh et al., 2002; Ma et al., 2004; Lu et al., 2008; D’Errico et al., 2009; Cho et al., 2011; Curtis et al., 2012), cardiovascular damage and remodeling (Tan et al., 2002; Mustonen et al., 2008; Cingolani et al., 2011; Frolova et al., 2012; Lynch et al., 2012) as well as liver regeneration (Klaas et al., 2016). In addition to its matricellular role, THBS4 is expressed in the central nervous system and found in blood serum (Gan and Sudhof, 2019; Yang et al., 2021). It can act on neurons as a synaptogenic factor (Gan and Sudhof, 2019) to protect from the loss of long-term memory (Yang et al., 2021).

Experiments with THBS4^–/–^ mice have shown that THBS4 promotes angiogenesis and skin wound healing by promoting endothelial cell adhesion, migration and proliferation (Muppala et al., 2015). Nevertheless, the regulation of THBS4 expression and the effects of THBS4 on cell behavior are rather poorly understood. TGF-β signaling has been proposed to mediate the angiogenetic properties of THBS4 (Muppala et al., 2017), however, the downstream signaling induced by THBS4 has received limited attention. TGF-β signaling targeted to THBS4 has been shown to be mediated by Smad3 (Muppala et al., 2017) and p38-MAPK, which proposedly increase THBS4 expression via a positive feedback loop (Qian et al., 2018). Recent studies with bladder cancer cells have shown that THBS4 is able to promote cancer progression by enhancing cell proliferation, migration and invasion by activating the AKT signaling pathway (Chou et al., 2021; Shi et al., 2021). THBS4 was also shown to bind integrin α2 to induce phosphorylation of heat shock factor 1, which increases TGF-β1 expression and promotes its paracrine signaling cascade (Shi et al., 2021).

Here we investigated the role of THBS4 in skin regeneration and elucidated its effects on cellular responses. We show that THBS4 expression is upregulated in healing skin wounds in humans and in mice. Using *in vitro* and *in vivo* methods, we demonstrate that application of recombinant THBS4 protein promotes wound healing and epithelialization. Furthermore, through proteotranscriptomic pathway analysis we demonstrate that THBS4 activates β-catenin signaling, which likely plays the key roles in promoting keratinocyte proliferation and fibroblast migration. Our results thus suggest that activating THBS4 signaling, for example by incorporating recombinant THBS4 into advanced wound care modalities, can offer new opportunities for the treatment of cutaneous wounds.

## Materials and Methods

### Mouse Wound Healing Experiments

Male 8-week-old C57/BL6 mice were used in the experiments. General anesthesia was induced by using 2–3% isoflurane in 100% oxygen (flow rate 1 L/min) and was maintained using 1% isoflurane. A 6-mm biopsy punch (Kai Medical, Solingen, Germany) was used to create full-thickness dermal wounds in the dorsal skin of mice; the skin removed by this method was considered as day 0 (healthy control) in this study. To avoid wound contraction, a silicone splint (2–3 mm wide, with an inner diameter of 6 mm) cut from a 0.5 mm thick silicone sheet (Grace Bio-Labs, Bend, OR, United States) was applied around the wound and fixed using a cyanoacrylate adhesive and surgical stitches. The wounds were covered with a transparent occlusive dressing (Tegaderm, 3M, Maplewood, MN, United States). In experiments testing the effect of THBS4 protein on the wound healing process, 1 μg of purified recombinant mouse THBS4 protein (R&D Systems, Minneapolis, MN, United States), product code 7860-TH-050) dissolved in 10 μl of phosphate buffered saline (PBS) was applied daily on the wounds. Equal volumes of PBS vehicle were applied to the wounds of the animals in the control group. The wounds were measured daily throughout the experiment. The mice were sacrificed at the specified time points and the skin samples from the healing wounds were embedded in O.C.T compound (Tissue-Tek, Sakura Finetek Europe B.V., Alphen aan den Rijn, the Netherlands) and stored at –80°C for further analysis. 10 μm-thick frozen sections were cut for immunofluorescence. All procedures involving animals were conducted according to the guidelines approved by the Commission of Laboratory Animal License at the Estonian Ministry of Agriculture (license no 180).

### Skin Burn Samples

The study involving burn injury patients was conducted according to Declaration of Helsinki principles and it has been approved by the Research Ethics Committee of the Helsinki University Hospital (DNro 101/E6/2000). Informed consent was obtained for all participants. Briefly, samples of 10 patients (age range 19–58 years), with large (total burn surface area range 22–45%) deep third-degree burns were used for immunofluorescence analysis. 3 mm punch biopsy samples were collected from the study area at 3, 14, and 21 days after the wound excision. Healthy normal skin was collected from healthy volunteers from similar locations that were not exposed to the sun. The samples were formalin-fixed and then embedded in paraffin. The paraffin-embedded tissue blocks were cut on microtome into 4.5-μm-thick sections that were mounted on Superfrost Plus slides (Thermo Fisher Scientific, Braunschweig, Germany). Deparaffinization and heat-induced antigen retrieval in 10 mM sodium citrate buffer, pH 6.0, were performed before immunofluorescence analysis.

### Psoriasis Samples

The adult patients with plaque psoriasis were recruited from the Tartu University Hospital at the Clinic of Dermatology between 2013 and 2015. This collection of tissue samples was approved by the Research Ethics Committee of the University of Tartu (permission number 245/M-18). The Declaration of Helsinki protocols were followed and patients gave their informed, written consent. 3 mm punch biopsies were taken from the well-defined psoriatic lesional skin from upper arm and torso of psoriasis patients as well as similar locations that were not exposed to the sun in controls. Tissue samples were embedded in O.C.T compound (Tissue-Tek, Sakura Finetek Europe B.V., Alphen aan den Rijn, the Netherlands) and stored at –80°C for further analysis. 10 μm-thick frozen sections were cut for immunofluorescence.

### Immunofluorescence Analysis

Tissue sections and cells grown on coverslips were fixed with 4% paraformaldehyde and permeabilized with 0.2% Triton X-100. After blocking with 5% normal donkey serum (Sigma-Aldrich, Merck Group, Darmstadt, Germany), the samples were incubated with primary antibodies overnight at + 4°C, followed by incubation with fluorochrome-conjugated secondary antibodies. Antibodies are listed in Supplementary Table 1. Nuclei were counterstained with DAPI (0.1 μg/ml, Thermo Fisher Scientific, Eugene, OR, United States).

### Generation of Thrombospondin-4 Expression Vector

To generate *THBS4*-expression plasmid, human *THBS4* sequence was amplified from ORF cDNA clone expression plasmid (catalog no HGI8843-UT, Sino Biological, Beijing, China). Amplified sequence was restricted with *Hin*dIII and *Xho*I restriction enzymes (Fermentas, Vilnius, Lithuania) and inserted into pcDNA3.1 expression vector (Thermo Fisher Scientific, Waltham, MA, United States). The cloning result was determined by sequencing (Institute of Genomics, University of Tartu, Tartu, Estonia).

### Cell Transfection

Human embryonic kidney cells HEK293 (ATCC #CRL-1573, obtained from the American Type Culture Collection, Manassas, VA, United States) were cultivated in Dulbecco’s Modified Eagle’s Medium (DMEM) (Gibco, Thermo Fisher Scientific, Paisley, United Kingdom) supplemented with 10% (v/v) fetal bovine serum (Corning Incorporated, Corning, NY, United States) and penicillin-streptomycin solution (Gibco, Thermo Fisher Scientific, Grand Island, NY, United States) resulting a final concentration of 100 units/ml penicillin and 100 μg/ml streptomycin. Prior to transfection the full media was replaced with DMEM containing 1X Insulin-Transferrin-Selenium supplement (Gibco, Thermo Fisher Scientific, Grand Island, NY, United States). HEK293 cells were transfected with 10 μg pcDNA3.1Zeo_THBS4 plasmid or empty vector control by using TurboFect reagent (Thermo Fisher Scientific, Vilnius, Lithuania) according to the manufacturer’s instructions. 48 h after the transfection the cells and cell media were collected for protein expression analysis (Supplementary Figure 1A) and for further use in cell culture experiments. A series of four dilutions (in triplicate), spanning the range 0–20 μg/ml, was used to prepare a calibration curve. The THBS4 concentration was calculated according to the regression line (Supplementary Figure 1B). Purified THBS4 and transfected HEK293 cell proteins were separated and visualized by Western blot method. Calibration curve was calculated according to the signal intensity of the purified THBS4 bands. Calculated THBS4 protein concentration in the 1 ml THBS-conditioned medium was 12 μg/ml. Since 25% dilution of conditioned medium in regular growth medium was used for *in vitro* experiments, the final concentration of THBS4 protein was 3 μg/ml.

### Fibroblast Culture

Human primary fibroblast culture was established by explant culture method. Briefly, skin from healthy donors (collected from breast reduction surgeries, Tartu University Clinics; ethics permit 292/T-4) was cut into small pieces and adhered to tissue culture dishes (Corning Incorporated, Corning, NY, United States). Skin pieces were covered in medium, that contained DMEM medium supplemented with 10% (v/v) fetal bovine serum (both Gibco, Thermo Fisher Scientific, Paisley, United Kingdom) and penicillin-streptomycin solution (Gibco, Thermo Fisher Scientific, Grand Island, United States) resulting a final concentration of 100 units/ml penicillin and 100 μg/ml streptomycin. Cells were allowed to migrate from dermis for 10–14 days. Cells from at least 3 patients were pooled and stored as frozen stocks for further experiments.

### Keratinocyte Culture

Skin from healthy donors (collected from breast reduction surgeries, Tartu University Clinics; ethics permit 292/T-4) was cut into small pieces and incubated in 2.4 U/ml dispase (Thermo Fisher Scientific, Grand Island, NY, United States) solution overnight at 4°C. The epidermis was separated and further dissociated using 0.05% trypsin solution (Thermo Fisher Scientific, Paisley, United Kingdom). The separated keratinocytes were cultured in EpiLife medium containing 1% human keratinocyte growth supplement (all Thermo Fisher Scientific, Grand Island, NY, United States) and penicillin-streptomycin solution (Gibco, Thermo Fisher Scientific, Grand Island, United States) resulting a final concentration of 100 units/ml penicillin and 100 μg/ml streptomycin. Pooled keratinocytes from 3 donors at passages 3–6 were used in experiments.

### Scratch Wound Healing Assay

Fibroblasts were seeded into wells of a 24-well plate (Corning Incorporated, Corning, NY, United States) and cultured until nearly confluent. Cells were stimulated with recombinant THBS4-containing medium (from transfected HEK293 cells, as described in section 1.6 Cell transfection) or control medium (from pcDNA mock-transfected HEK293 cells), respectively, for 24 h before the scratch assay. Scratches were made using a sterile 1 ml pipette tip. The wound width was imaged under the Nikon Eclipse TS100 microscope (Nikon Instruments, Melville, NY, United States) and the photographs captured using a digital camera head (DS-Vi1, Nikon) and a stand-alone controller and display unit (DS-L3, Nikon). Images were captured immediately after creating the wounds (timepoint 0) and every 3 h thereafter. The wound width was then measured from the images using ImageJ software (Schneider et al., 2012) and the wound closure percentages were calculated.

### Transwell Migration Assay

For 24 h before the start of the migration assay, the fibroblasts were stimulated with THBS4-conditioned medium (from transfected HEK293 cells) or control medium (from pcDNA mock-transfected HEK293 cells) in 1:4 ratio. A total of 1.5 × 10^4^ cells were seeded in serum-free medium on the upper membrane of the transwell chamber (6.5 mm transwell with 8.0 μm pore polycarbonate membrane insert, Corning Incorporated, Kennebunk, ME, United States). Cells were allowed to migrate for 24 h and then fixed and stained with 0.5% Coomassie Brilliant Blue G-250 (Sigma-Aldrich). The cells that did not migrate through the membrane were removed with a moist cotton swab. Pictures were taken from 6 different places of each well under a 10x objective lens of Nikon Eclipse TS100 microscope (Nikon Instruments, Melville, NY, United States) equipped with a digital camera head (DS-Vi1, Nikon) and a stand-alone controller and display unit (DS-L3, Nikon). The cells that migrated through the membrane were quantified per each field of view.

### RNA Isolation

Fibroblasts were grown on 60 mm-diameter cell culture dishes (Corning Incorporated, Corning, NY, United States) until 70–80% confluency and then stimulated with THBS4-conditioned medium or control medium (from pcDNA mock-transfected HEK293 cells) in 1:4 ratio for 4 h. Total RNA was separated using the RNeasy Mini Kit (Qiagen, Hilden, Germany) according to the manufacturer’s instructions. Separated RNA was processed further for RNA-sequencing.

### RNA-Sequencing Analysis

RNA-sequencing analysis and read mapping was performed as a service at EMBL Genomics Core Facility (Heidelberg, Germany). The Galaxy platform^1^ (Afgan et al., 2018) was used for transcriptomics analysis. RNA STAR (Galaxy version 2.7.7a) was used for sequence alignment to reference genome GRCh38/h38.87 and featureCounts (Galaxy version 2.0.1) was used to count the number of reads. Differential expression analysis was conducted using the DESeq2 package (Love et al., 2014). Data was uploaded to Gene Expression Omnibus^2^ (GEO, accession no. GSE179969). Gene was considered differentially expressed if the adjusted *P* < 0.05.

### Proteomics Analysis

Fibroblasts were grown on 100 mm-diameter cell culture dishes (Falcon Corning, Corning, NY, United States) until 70–80% confluency and then stimulated with THBS4-conditioned medium or control medium (from pcDNA mock-transfected HEK293 cells) in 1:4 ratio for 24 h. Cells were lysed in an NP40 buffer containing protease inhibitors (Halt Protease Inhibitor Cocktail, Thermo Fisher Scientific, Rockford, IL, United States). For the full proteome analysis, 5 μg of protein was precipitated with 100% (w/v) Trichoroacetic Acid/Sodium Deoxycholate solution. Next, proteins were reduced, alkylated and digested by Lys-C protease and trypsin. The peptides were separated on a Ultimate 3000 RSLCnano system (Dionex, Sunnyvale, CA, United States) using a C18 cartridge trap-column in a backflush configuration and an in-house packed (3 μm C18 particles, Dr. Maisch, Ammerbuch, Germany) analytical 50 cm × 75 μm emitter-column (New Objective, Woburn, MA, United States). Separated peptides were eluted to a quadrupole-orbitrap Q Exactive Plus (Thermo Fisher Scientific, Waltham, MA, United States) tandem mass spectrometer (MS) operating with a top-10 data dependent acquisition strategy. Raw data were processed with the MaxQuant software package and UniProt^3^ database using the tryptic digestion rule. See Supplementary Methods for further details. The mass spectrometry proteomics data have been deposited to the ProteomeXchange Consortium via the PRIDE (Perez-Riverol et al., 2019) partner repository with the dataset identifier PXD027364.

### Ingenuity Pathway Analysis

Pathway analyses were performed using a similar workflow as reported earlier (Xie et al., 2020). Briefly, differentially expressed gene (DEG) and protein (DEP) data were imported into IPA Ingenuity Pathway Analysis software (Qiagen, Version 62089861). Significant pathways were compared and pathways with the major overlaps in RNA sequencing and proteomics data were selected for further analysis. Through filtering, a single common gene/protein (H4C1, H4 Clustered Histone 1) was found to be differentially expressed in both transcriptomics and proteomics data. In IPA, a H4C1-centered network was expanded fully and limited on those DEGs and DEPs in the datasets including direct and indirect interactions both up- and downstream. Expression values were overlaid and when applicable were complemented with both up- and downstream effects predicted using IPA’s Molecule Activity Predictor. An overlay graphic of the H4C1-centered graph to include both DEPs and DEGs was compiled using Inkscape software (Inkscape 1.0.1, Scalable Vector Graphics Editor).

### Data Analysis and Statistics

Statistical significance was determined by one-way ANOVA followed by Tukey’s post-test (multiple comparisons) or Student’s *t*-test (two groups). *P*-values < 0.05 were considered significant.

## Results

### Thrombospondin-4 Is Upregulated in Wound Healing and in Psoriatic Lesions

**FIGURE 1:**
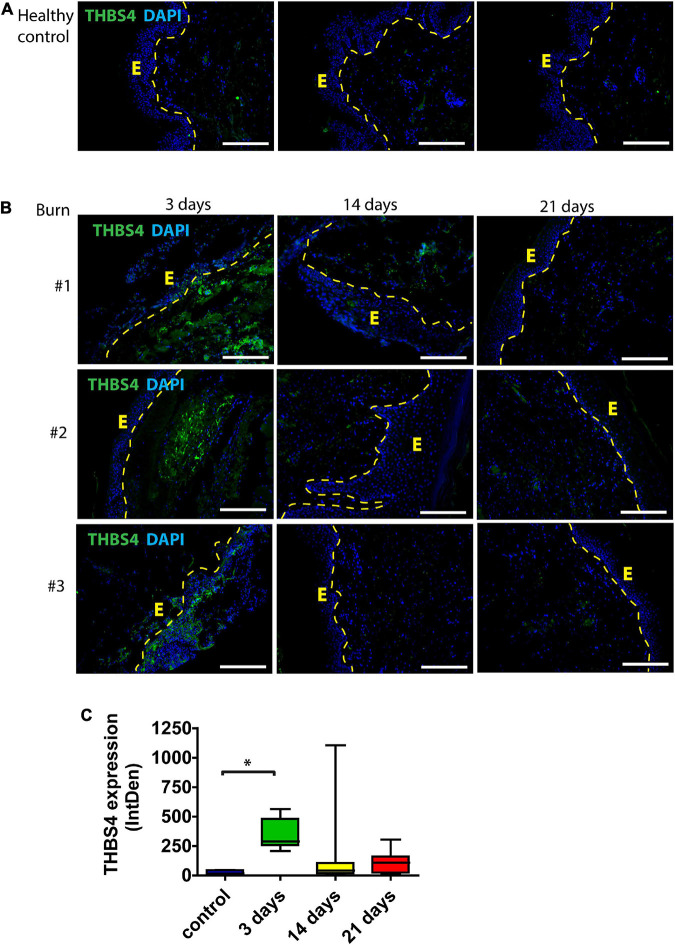
THBS4 expression in human skin following burn injury. **(A)** Normal skin from healthy controls; **(B)** skin from burn injury patients. Biopsy samples were collected from the study area at 3, 14, and 21 days after the wound excision. E—epidermis. 3 representative samples in each group are shown. Scale bar is 200 μm. **(C)** Relative quantification of THBS4 expression by mean integrated density of the fluorescence signal. The plot depicts the distribution of 10 samples, * indicates a statistically significant (*P* < 0.05) difference.

First, we sought to characterize the expression of THBS4 protein in normal human skin and in healing cutaneous wounds. Immunofluorescence analysis showed that in normal healthy skin THBS4 expression was very low or undetectable (Figure 1A). In contrast, the expression of THBS4 was detected at high levels in healing skin areas of burn patients with the strongest expression at 3 days following excision and skin grafting of the burn injury (Figure 1B) with the maximal THBS4 expression in the ECM of dermis near the healing wound bed. Quantification of the THBS4 expression showed that in later time points (14, 21 days post operation of the burn injury) THBS4 expression was more than twofold reduced, indicating that the increase in THBS4 is induced mainly in the proliferative phase of skin wound healing (Figure 1C).

**FIGURE 2:**
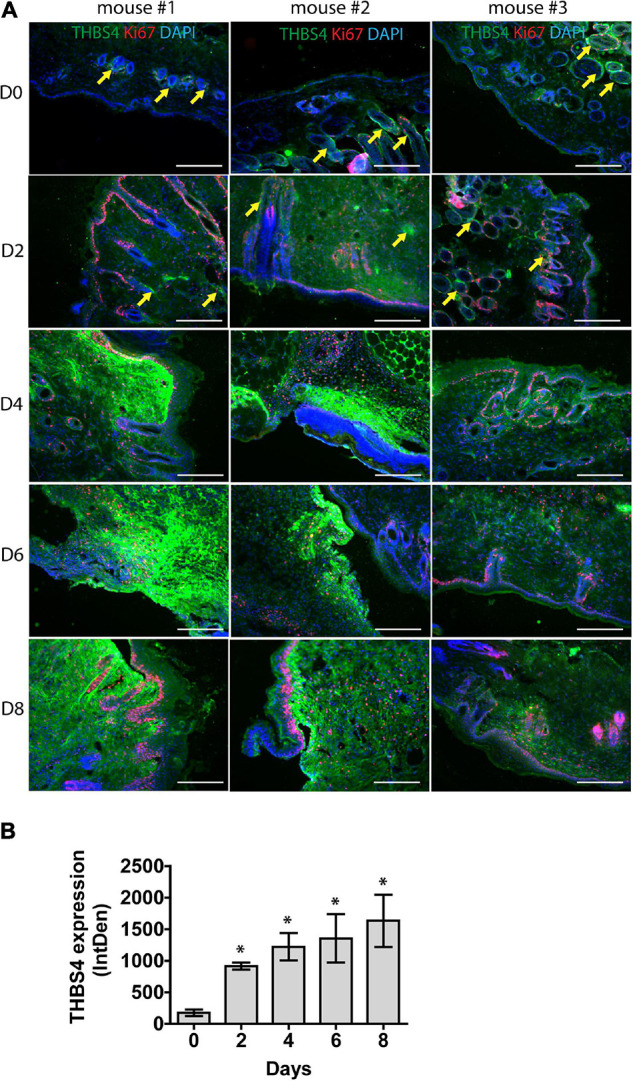
THBS4 is upregulated in regenerating mouse skin. Full-thickness dermal wounds were generated in mouse dorsal skin. **(A)** THBS4 expression was characterized by immunofluorescence microscopy in healthy skin and at 2-, 4-, 6-, and 8-days post wounding. 3 representative samples in each group are shown. Yellow arrows indicate THBS4 expression in hair follicles. Scale bar is 200 μm. **(B)** Relative quantification of THBS4 expression by mean integrated density of the fluorescence signal. Bars show the average of 3 samples for each time point ± standard deviation, * indicates a statistically significant (*P* < 0.05) difference.

To further characterize THBS4 expression in acute wound healing, we utilized an *in vivo* mouse model of full-thickness excisional wounds (Figure 2). Similarly to human skin, very low levels of THBS4 were detected in healthy mouse skin, with the exception of hair follicles which showed high expression of THBS4 in the outer root sheath (Figure 2A). On average, a fivefold upregulation of THBS4 was observed in dermis of regenerating skin at day 2 post wounding (Figure 2B). The strongest expression of THBS4 was detected at days 4–8 of wound healing in the vicinity of proliferating Ki67^+^ basal epidermal keratinocytes (Figure 2) where 7–9-fold upregulation of THBS4 expression was observed (Figure 2B).

**FIGURE 3:**
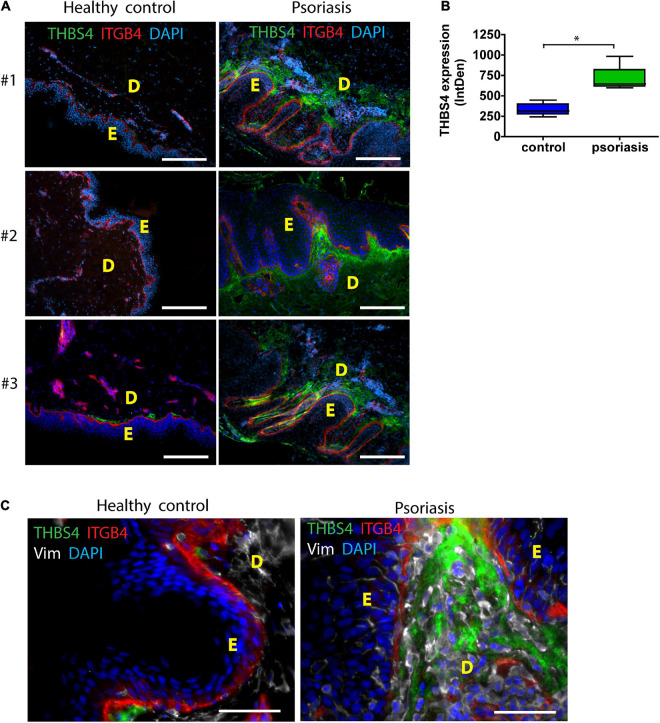
THBS4 expression in healthy control skin and psoriatic skin lesions. E—epidermis; D—dermis. 3 representative samples in each group are shown **(A)** and relative quantification of THBS4 expression by mean integrated density of the fluorescence signal **(B)**, *n* = 5. Scale bar is 200 μm. **(C)** THBS4 co-localization with fibroblast marker vimentin (Vim) and integrin beta 4 (ITGB4). Scale bar is 50 μm. The plot depicts the distribution of 5 samples, * indicates a statistically significant (*P* < 0.05) difference.

Psoriasis is a chronic inflammatory skin disorder that is characterized by hyperproliferative skin lesions (Griffiths et al., 2021). As it is known that wounds heal faster in psoriatic skin (Morhenn et al., 2013), we analyzed THBS4 expression in psoriatic lesions (Figures 3A,B). We found that THBS4 was upregulated in psoriatic skin lesions by more than 2-fold (Figure 3B). Double staining with integrin β 4 (ITGB4), a marker of basal epidermal cell layer revealed a juxtaposition of THBS4-high papillary dermis and basal keratinocyte layer where keratinocyte proliferation takes place (Figures 3A,C) suggestive of a positive impact of THBS4 on keratinocyte proliferation in psoriatic skin lesions. A detailed examination of the psoriatic skin lesions revealed that the strongest THBS4 signal localizes inside and in the vicinity of the active vimentin^+^ fibroblasts under *rete ridges* of the epidermis (Figure 3C and Supplementary Figure 2).

### Thrombospondin-4 Supports Fibroblast Migration but Not Proliferation

Since the most dramatic THBS4 upregulation was detected in dermis during wound healing, we aimed to further characterize the effect of THBS4 on skin fibroblasts, the main cell type present in the dermal compartment of the skin.

To perform the *in vitro* experiments, we used THBS4-enriched medium harvested from HEK293 cells transfected with the corresponding expression plasmid as large quantities of protein were needed. The use of THBS4-conditioned medium may incur inadvertent effects arising from the presence of constitutively secreted proteins from the host cell line as well as those induced by the transfection procedure. To minimize these effects, we used the supernatant harvested from the parallel HEK293 cultures that were transfected with the empty expression vector as a control.

**FIGURE 4:**
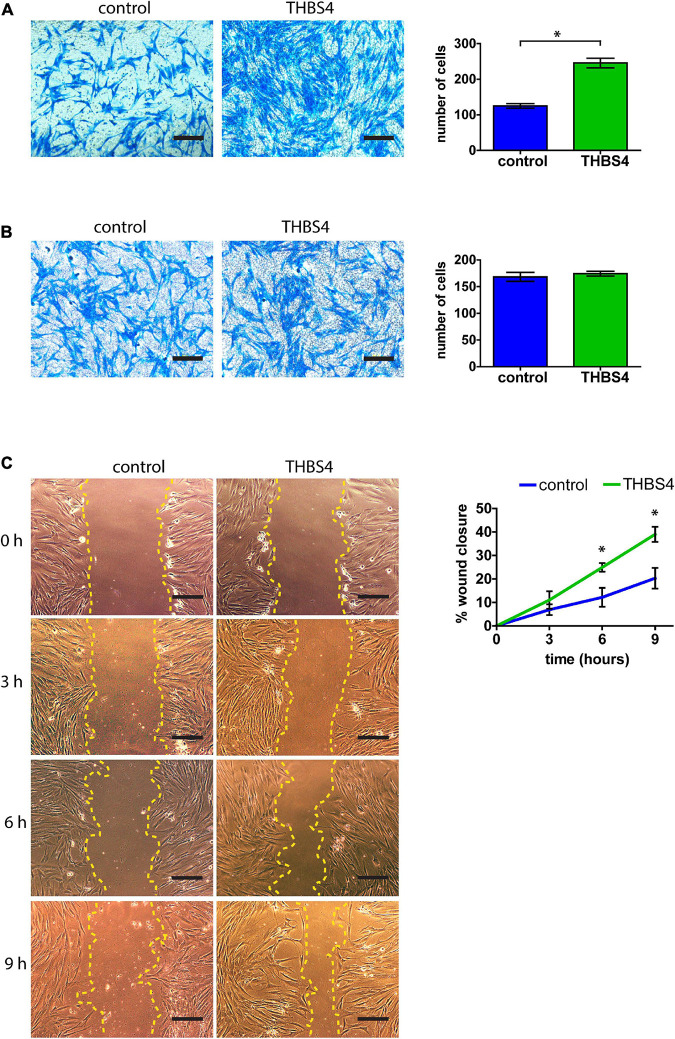
THBS4 promotes fibroblast migration. **(A)** Transwell migration assay with fibroblasts stimulated with recombinant THBS4 and quantification of the number of migrating cells in a field of view. **(B)** Transwell invasion assay through Matrigel-coated chambers and quantification of the number of migrating cells in a field of view. **(C)** Representative images of the *in vitro* scratch wound healing assay with fibroblasts stimulated with recombinant THBS4 and quantification of the relative wound closure in time. Scale bar is 200 μm. The graphs depict the averages of at least 3 independent replicates ± standard deviation, * indicates a statistically significant (*P* < 0.05) difference compared to cells stimulated with control medium.

**FIGURE 5:**
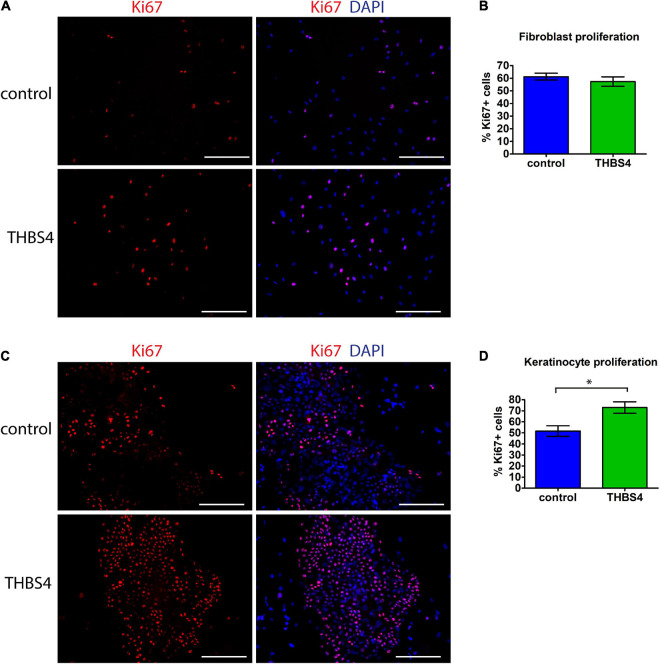
THBS4 promotes keratinocyte but not fibroblast proliferation *in vitro*. **(A)** Representative images of primary human fibroblasts cultured in the presence of recombinant THBS4 protein and the quantification of Ki67^+^ positive cells, *n* = 3 **(B)**. **(C)** Representative images of primary human keratinocytes cultured in the presence of recombinant THBS4 protein and quantification of Ki67^+^ positive cells. **(D)** Scale bar is 200 μm. The graphs depict the averages of at least 3 independent replicates ± standard deviation, * indicates a statistically significant (*P* < 0.05) difference compared to cells stimulated with control medium.

Primary human fibroblasts cultivated in THBS4-supplemented media (see Supplementary Figure 1 for details) showed a significantly increased migration rate in the transwell migration assay when compared to fibroblasts cultured in control media (Figure 4A). On average, THBS4-stimulated cells were able to migrate 1.97 times more effectively during the 24-h experiment than the control cells (Figure 4A). However, no significant differences were detected in cell invasiveness using Matrigel-coated transwell assay between cells cultivated in THBS4-supplemented and in control media (Figure 4B). An improved cell migration upon THBS4 stimulation was also observed in an *in vitro* scratch assay (Figure 4C). A significantly faster void closure (*p* = 0.045) was detected at 6 h where THBS4-stimulated cells had recovered 24.9 ± 3.2% of the initial defect while the control cells had recovered only 12.2 ± 6.9%. Improved scratch recovery was maintained at 9 h time point where fibroblasts cultivated in the presence of THBS4 showed nearly 2 times faster void closure on average, *p* = 0.033 (Figure 4C). To investigate whether THBS4 could stimulate the proliferation of the main cellular components of skin—fibroblasts and keratinocytes, we cultivated primary human skin cells in the presence of recombinant THBS4 in the cell culture medium (Figure 5). Immunofluorescence analysis of the proliferation marker Ki67 expression revealed that there was no change in the proliferation rate of fibroblasts when the cells were cultured in the presence of recombinant THBS4 protein (Figures 5A,B). Interestingly, the proliferation of keratinocytes was considerably increased when cultivated in the presence of THBS4, *p* = 0.0237 (Figures 5C,D).

### The Effect of Thrombospondin-4 Stimulation on Fibroblast Proteotranscriptomic Profile

**FIGURE 6:**
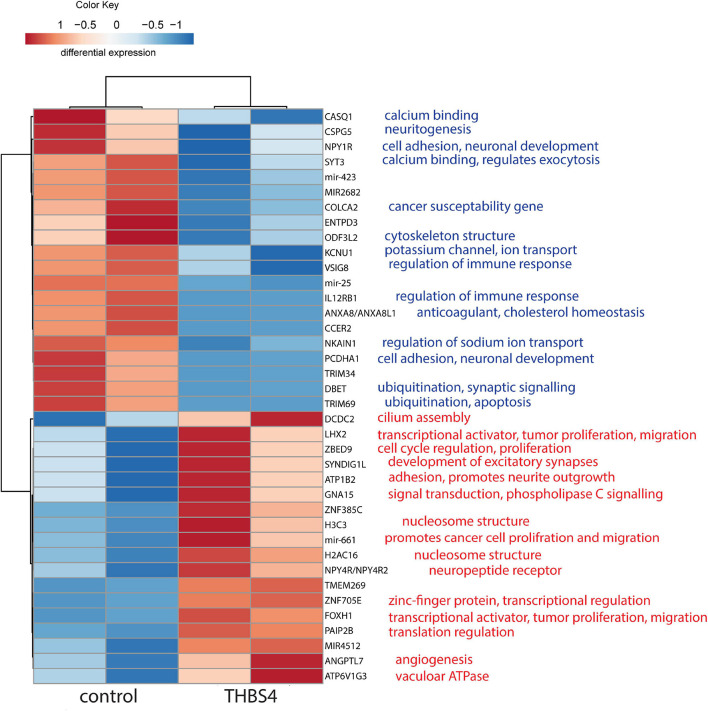
Heatmap of differentially enriched genes in fibroblasts in response to THBS4 stimulation. Statistically significant (*P* < 0.05) and expression fold-change > 2 protein coding genes and microRNAs are shown, *n* = 2.

To understand the molecular mechanisms by which THBS4 increases fibroblast migration, we first compared the transcriptional profile of fibroblasts either grown in the presence of THBS4 or in control media (Figure 6). We detected a 7.4-fold upregulation of Angiopoietin Like 7 (ANGPTL7), which is a known pro-angiogenetic factor (Parri et al., 2014), and 3.7-fold upregulation of transcriptional factor forkhead box H1 (FOXH1), which has been shown to activate Wnt/β-catenin signaling pathway and promote cancer cell proliferation and migration (Zhang et al., 2021). Moreover, we detected upregulation of several other transcriptional regulators that have been associated with cell proliferation and migration: ZBED9 (5.2-fold upregulation), LHX2 (2.4-fold) and mir-661 (10.5-fold upregulation). On average, 4.9-fold down-regulation of neuropeptide Y receptor Y1 (NPY1R) was observed, which has been shown to inhibit the proliferation, migration and invasiveness of cancer cells (Lv X. et al., 2016; Li et al., 2020). The expression of ubiquitination and apoptosis-associated factors DBET and TRIM69 were downregulated by 2.2- and 7.0-fold, respectively. Downregulation of immunoregulatory factors IL12RB1 (6.0-fold) and VSIG8 (6.1-fold) was also observed.

**FIGURE 7:**
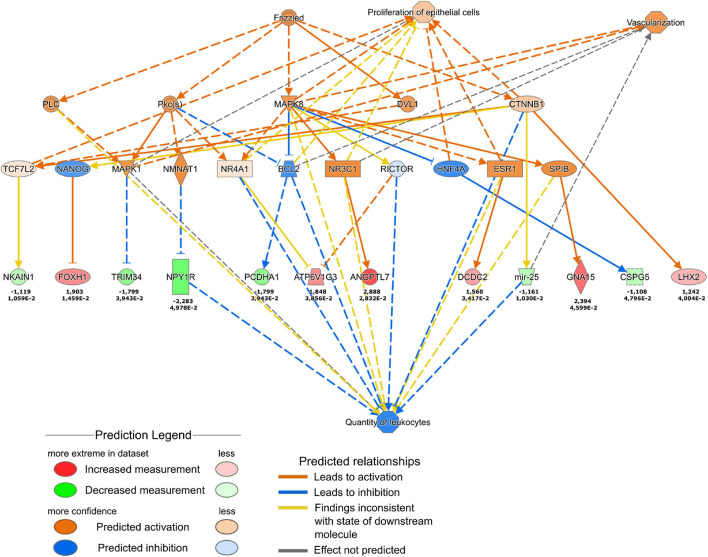
Ingenuity Pathway Analysis of the changes in fibroblast transcription profile in response to THBS4 stimulation. Causal network showing Frizzled and β-catenin 1 pathway activation is shown. Upper values indicate log2 fold changes, lower values show the *P*-value for the RNAseq.

More refined analysis of the cell transcriptome using Qiagen Ingenuity Pathway analysis (Figure 7) suggested the activation of the WNT pathway. More specifically, the results predicted the increased Wnt signaling via Frizzled and β-catenin 1 (CTNNB1) that were predicted to promote vascularization and the proliferation of cells. The downstream effects of the Frizzled-CTNNB1 signaling are likely to support the reduction of the quantity of leukocytes which could promote the transition from the inflammatory to the proliferative phase in the wound healing process. Interestingly, the in-depth analysis revealed the downregulation of genes belonging to the interleukin-12 (IL-12) pathway (Supplementary Figure 3A), that is involved in regulation of wound healing (Matias et al., 2011). The predicted downregulation of stress-inducible transcription regulator NUPR1 (Supplementary Figure 3B) is likely to inhibit apoptosis in fibroblasts.

To further characterize the downstream effects of THBS4 stimulation we carried out proteomic profiling of THBS4-stimulated fibroblasts. Analysis of upregulated proteins by THBS4 treatment revealed that the majority of the proteins were associated with mitochondria and adhesion (Supplementary Figure 4A) that indicated that THBS4 strongly stimulated subcellular mechanisms that have crucial roles in cell migration. Pathway analysis of the THBS4-enriched proteins revealed the strongest enrichment in proteins that play a role in epithelial to mesenchymal transition, regulation of apoptosis and DNA repair (Supplementary Figure 4B and Supplementary Table 2), all the processes that could significantly contribute to the wound healing process.

**FIGURE 8:**
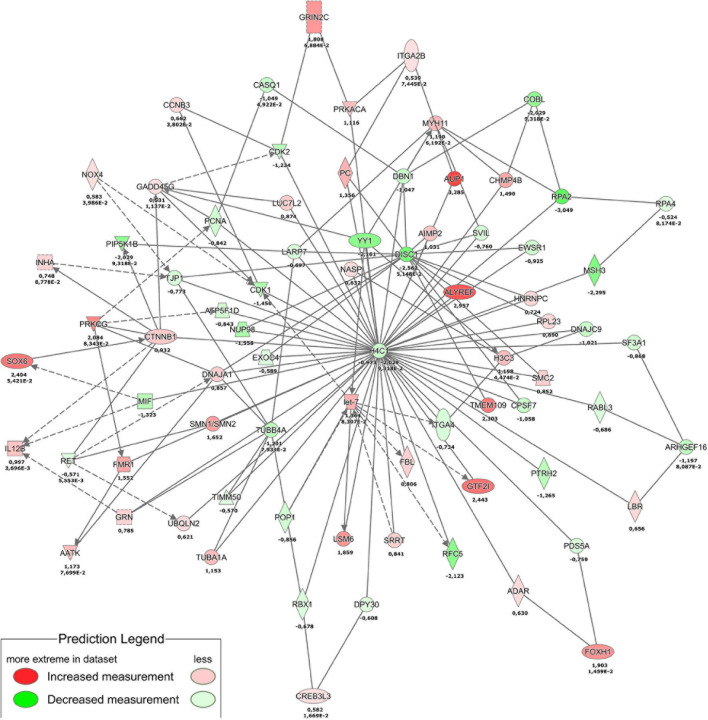
Proteotranscriptomic analysis of THBS4 stimulation in fibroblasts. The combined network of transcriptomics analysis at 4 h stimulation and proteomics analysis at 24 h is shown. Single numbers indicate log2FC in proteomics and duplicate values indicate log2FC and *P*-value for RNAseq.

Combined analysis of transcriptomics and proteomics (Figure 8) revealed histone H4 (H4C1) downregulation central in the THBS4-induced signaling network. Downregulation of histone H4 transcription has previously been implicated in the onset of cell differentiation (Gerbaulet et al., 1992), which could also play a role in fibroblast differentiation into motile myofibroblasts. Causal network combined pathway analysis of RNA-sequencing and proteomics analysis (Supplementary Figure 5) identified activated common pathways upon THBS4-stimulation that were again centered on β-catenin (CTNNB1). Gene expression from RNA sequencing data predicted CTNNB1 activation which was also found upregulated in proteomic analysis (Supplementary Figure 5). In line with the proteomic data we found a 1.7–2.1-fold increase of β-catenin expression in fibroblasts stimulated with THBS4 for 24 h using Western blot analysis (Supplementary Figures 6A,B) and immunofluorescence microscopy analysis (Supplementary Figures 6C,D). In addition, we analyzed the expression of β-catenin in human healing burn wounds. We found that β-catenin levels were significantly upregulated in the dermal areas with high THBS4 content (Supplementary Figure 7). However, no significant changes in β-catenin expression in the dermis of psoriasis patients was found (Supplementary Figure 8).

### Thrombospondin-4 Promotes Wound Healing *in vivo*

**FIGURE 9:**
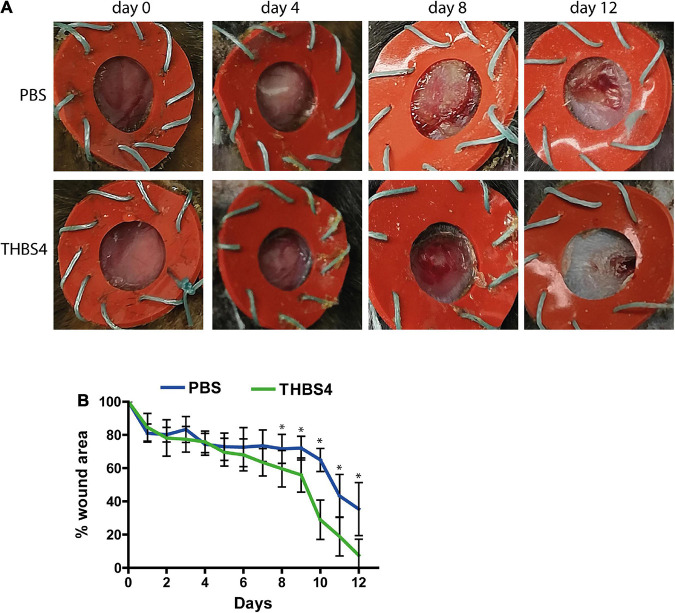
THBS4 promotes wound healing *in vivo*. Full dermal wounds were inflicted on the dorsal skin of C57/Bl6 mice and either recombinant THBS4 or PBS was applied. Representative images of the wounds from indicated time points **(A)** and measurements of the wound area **(B)**. The graph depicts the averages of 10 biological replicates ± standard deviation, * indicates a statistically significant (*P* < 0.05) difference.

Finally, we aimed to investigate whether providing external THBS4 promoted the healing of cutaneous wounds *in vivo* using the mouse excisional wound splinting model (Figure 9). The solution containing 1 μg of recombinant THBS4 protein was applied on wounds daily and the healing rate was measured for 12 days (Figure 9A). Compared to control (PBS-treated) wounds, the recombinant THBS4-treated wounds showed significantly improved healing rates from day 8 onward (Figure 9B). The strongest effect was observed at the final 12-day time point where only 7.5% of the wound area was open on average in the THBS4-treated group, whereas in PBS-treated controls the residual wound area was 35.4% on average.

## Discussion

Skin regeneration is a complex process that relies on several cellular responses and extracellular factors (Rousselle et al., 2019). In this work we demonstrated that THBS4 was an important contributor to cutaneous wound healing by stimulation of fibroblast migration at least in part via activation of β-catenin-dependent signaling pathways. As a proof of principle, we showed that the application of recombinant THBS4 directly to the wound area increased significantly cutaneous wound healing in a mouse full-thickness splinted wound model. These results highlight the potential benefits of incorporation of THBS4 protein in future wound healing therapies.

To our knowledge, this is the first study to report the upregulation of THBS4 expression in regenerating human skin. Previous studies with THBS4^–/–^ mice have demonstrated decreased wound healing when THBS4 was lacking (Muppala et al., 2015), however, this effect was mostly attributed to its angiogenic properties mediated by TGF-β1. This view was backed by the fact that THBS4 expression was increased in hypertrophic scar fibroblasts suggesting that THBS4 was a target of TGF-β1-dependent fibrotic response (Qian et al., 2018). In contrast, here we showed that THBS4 expression was rapidly increased in dermal ECM already in the early, proliferative phase of wound healing. Similarly, THBS4 was increased in the hyperproliferative skin of psoriasis patients where no fibrotic changes are present. As TGF-β is a critical regulator of different phases of wound healing (Gilbert et al., 2016), it is very likely that this early upregulation of THBS4 expression is also mediated through TGF-β signaling in fibroblasts but does not necessarily involve a fibrotic response.

**FIGURE 10:**
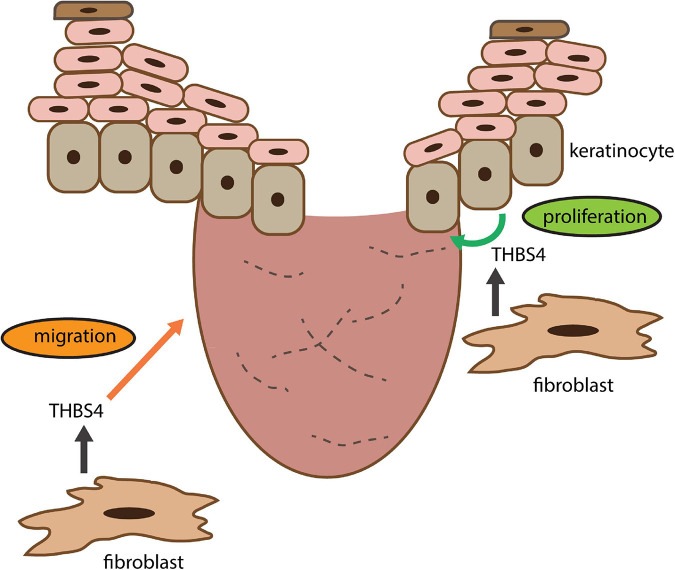
THBS4 is a soluble dermal inflammatory signal that activates the fibroblast migration for skin regeneration and wound healing. See section “Discussion” for closer explanation.

Previous studies have shown that upregulation of THBS4 expression in fibroblasts through viral transfection can increase the expression of TGF-β1 and α-SMA (Qian et al., 2019) suggesting a potential role for a positive feedback loop in regulation of THBS4 expression. Therefore, it is possible that in wound healing THBS4 upregulation in the dermis initiates a positive feedback loop that stimulates fibroblast migration to the wound bed and promotes wound healing via newly produced THBS4-containing ECM that supports keratinocyte proliferation and re-epithelization (Figure 10). Interestingly, we were not able to detect upregulation of TGF-β–related pathways in fibroblasts stimulated with extracellular THBS4 suggesting that a whole organism context may be required for the fully functional THBS4-TGF-β regulative axis.

Our *in vitro* experiments showed that external stimulation of fibroblasts with soluble THBS4 increases their migration but not proliferation. Interestingly, stimulation of keratinocytes with THBS4 yielded upregulation of their proliferation that well correlated with the hyperproliferative state of keratinocytes in psoriatic lesions that dwell in the vicinity of ECM containing abnormally high levels of THBS4. Nevertheless, THBS4 has been shown to specifically activate the migration of various cell types before, including other mesoderm-derived cells such as smooth muscle cells of different origin (Frolova et al., 2010; Girard et al., 2014; Lv X. et al., 2016; Su et al., 2017; Andres Sastre et al., 2021; Chou et al., 2021). Interestingly, our results on skin cell-type-selective responses to THBS4 stimulation are supported by previous studies on other tissues and cell types. For example, Andres Sastre and colleagues reported recently that THBS4 stimulated endothelial cell but not bone marrow stromal cell migration (Andres Sastre et al., 2021). Studies with THBS4^–/–^ mice have shown that THBS4 does not affect neuronal cell proliferation but in contrast, promotes their migration (Girard et al., 2014). In two recent studies THBS4 promoted cancer progression by enhancing tumor cell migration and invasion (Su et al., 2017; Chou et al., 2021).

Of note, THBS4 expression can also be upregulated in macrophages (Zhou et al., 2014; Lv L. et al., 2016; Rahman et al., 2020) that have a significant role in the wound healing process.

Furthermore, THBS4 produced by pro-inflammatory mouse macrophages supports their accumulation and inflammatory activity in an autocrine manner in an LPS-induced peritonitis model suggesting an immunomodulatory role for THBS4 in inflammation and potentially in wound healing (Rahman et al., 2020).

The precise signal transduction mechanism from THBS4 to intracellular effectors is not yet clear, as only a few cell surface molecules are known to bind to and mediate signaling from THBS4. Previous studies have established that THBS4 is involved in several pathological processes where it promotes cell proliferation through integrin binding (Frolova et al., 2010; Muppala et al., 2015; Shi et al., 2021). It was shown that THBS4 enhanced the proliferation of endothelial cells through binding with integrin ITGA2 (Muppala et al., 2015). ITGA2 is expressed in epidermal keratinocytes as well as in dermal fibroblasts and its presence is required for wound closure in human but not in mouse skin (Chen et al., 1999; Dumin et al., 2001; Zweers et al., 2007). Remarkably, a recent study in a gallbladder tumor model (Shi et al., 2021) demonstrated that THBS4 produced by fibroblasts enhances cancer cell proliferation through ITGA2 binding on target cells attributing a role for THBS4-ITGA2 interaction in regulating cell behavior. The proliferation of keratinocytes could also be mediated through indirect interaction between EGF-like repeats of THBS4 and EGFR present on these cells as both THBS1 and THBS4 were able to activate the autophosphorylation of EGFR subunits (Liu et al., 2009). Interestingly, no direct binding of THBS1 was observed, however, the activity of a metalloprotease was required for the signaling event to take place.

Apart from activating intracellular signaling cascades directly, THBS4 may exert its cellular effects via indirect mechanisms. The C-terminal domains of THBS4 bind to both collagenous and non-collagenous ECM proteins (Narouz-Ott et al., 2000) that could support fibroblast migration in dermis. Fibroblasts play a crucial role in skin regeneration starting from the inflammatory phase until final epithelization of the wound bed by secreting ECM proteins as well as soluble growth factors and cytokines (Bainbridge, 2013; Maddaluno et al., 2017).

Analysis of the fibroblast transcriptional profile in response to THBS4 stimulation revealed upregulation of angiogenic factor ANGPTL7, which could promote the formation of new blood vessels and their invasion into the wound bed corroboration previous findings that suggest a role for THBS4 in angiogenesis. The downregulation of the ubiquitination pathway and reduced expression of the apoptosis-associated DBET and TRIM69 in THBS4-stimulated fibroblasts suggest that THBS4 may promote fibroblast survival. Increased migration of fibroblasts in response to THBS4 stimulation is likely mediated through upregulation of transcription factor FOXH1 that has been shown to promote cancer cell proliferation and migration via activation of Wnt/β-catenin signaling pathway (Zhang et al., 2021). Additionally, the activation of integrin signaling via binding of THBS4 to ITGA2 may contribute to activation of Wnt/β-catenin pathway as ITGA2 has been shown to be instrumental for platelet-induced activation of Wnt/β-catenin signaling in MCF7 breast carcinoma cells (Zuo et al., 2019). The active role of THBS4 in regulation of cell motility and proliferation was further supported by the downregulation of NPY1R, which was shown to inhibit cancer cell proliferation, migration and invasiveness (Lv X. et al., 2016; Li et al., 2020).

Interestingly, we found downregulation of genes related to IL-12 signaling. In line with our data the loss of IL-12/23 function in knock-out mice enhanced cutaneous and mucosal wound healing (Matias et al., 2011).

Analysis of the changes in fibroblast proteome in response to THBS4 treatment showed a strong enrichment of mitochondria and adhesion-associated proteins, which act as important contributors to cell migration and invasion (Denisenko et al., 2019; Janiszewska et al., 2020). Pathway analysis of the proteome of THBS4-stimulated fibroblasts well corroborated the findings of transcriptome analysis by demonstrating the central role of β-catenin in regulating the detected changes in protein levels.

Taken together, we showed that THBS4 is upregulated in healing skin wounds and its supplementation activates the migratory properties of cutaneous fibroblasts via activating several cellular pathways that were partially coordinated by the central regulator of Wnt signaling—β-catenin 1. We conclude that the introduction of THBS4 into wound treatment modalities may offer novel opportunities for treatment of cutaneous wounds as demonstrated by the enhancement of wound healing *in vivo* in mice.

## Data Availability Statement

The RNA sequencing data were uploaded to Gene Expression Omnibus (https://www.ncbi.nlm.nih.gov/geo/) (GEO accession no GSE179969). The proteomics data are available via ProteomeXchange with identifier PXD027364.

## Ethics Statement

The studies involving human participants were reviewed and approved by the Research Ethics Committee of the Helsinki University Hospital. The patients/participants provided their written informed consent to participate in this study. The animal study was reviewed and approved by the Commission of Laboratory Animal License at the Estonian Ministry of Agriculture.

## Author Contributions

MK, KM-A, and EH performed the experiments. EK and CC-L analyzed the transcriptomics data. KK, ME, TA, EK, and HL provided human tissue samples. MK and VJ conceived the study, analyzed the data, and wrote the manuscript. All authors reviewed the results and approved the final version of the manuscript submitted for publication.

## Conflict of Interest

The authors declare that the research was conducted in the absence of any commercial or financial relationships that could be construed as a potential conflict of interest.

## Publisher’s Note

All claims expressed in this article are solely those of the authors and do not necessarily represent those of their affiliated organizations, or those of the publisher, the editors and the reviewers. Any product that may be evaluated in this article, or claim that may be made by its manufacturer, is not guaranteed or endorsed by the publisher.

## Footnotes

1
https://usegalaxy.eu/

2
https://www.ncbi.nlm.nih.gov/geo/

3
www.uniprot.org
