# Supplementary figures and images for: Thrombospondin-4 Is a Soluble Dermal Inflammatory Signal That Selectively Promotes Fibroblast Migration and Keratinocyte Proliferation for Skin Regeneration and Wound Healing
Source: Front Cell Dev Biol. 2021 Sep 23;9:745637. doi: 10.3389/fcell.2021.745637 (PMC8495264; doi:10.3389/fcell.2021.745637)

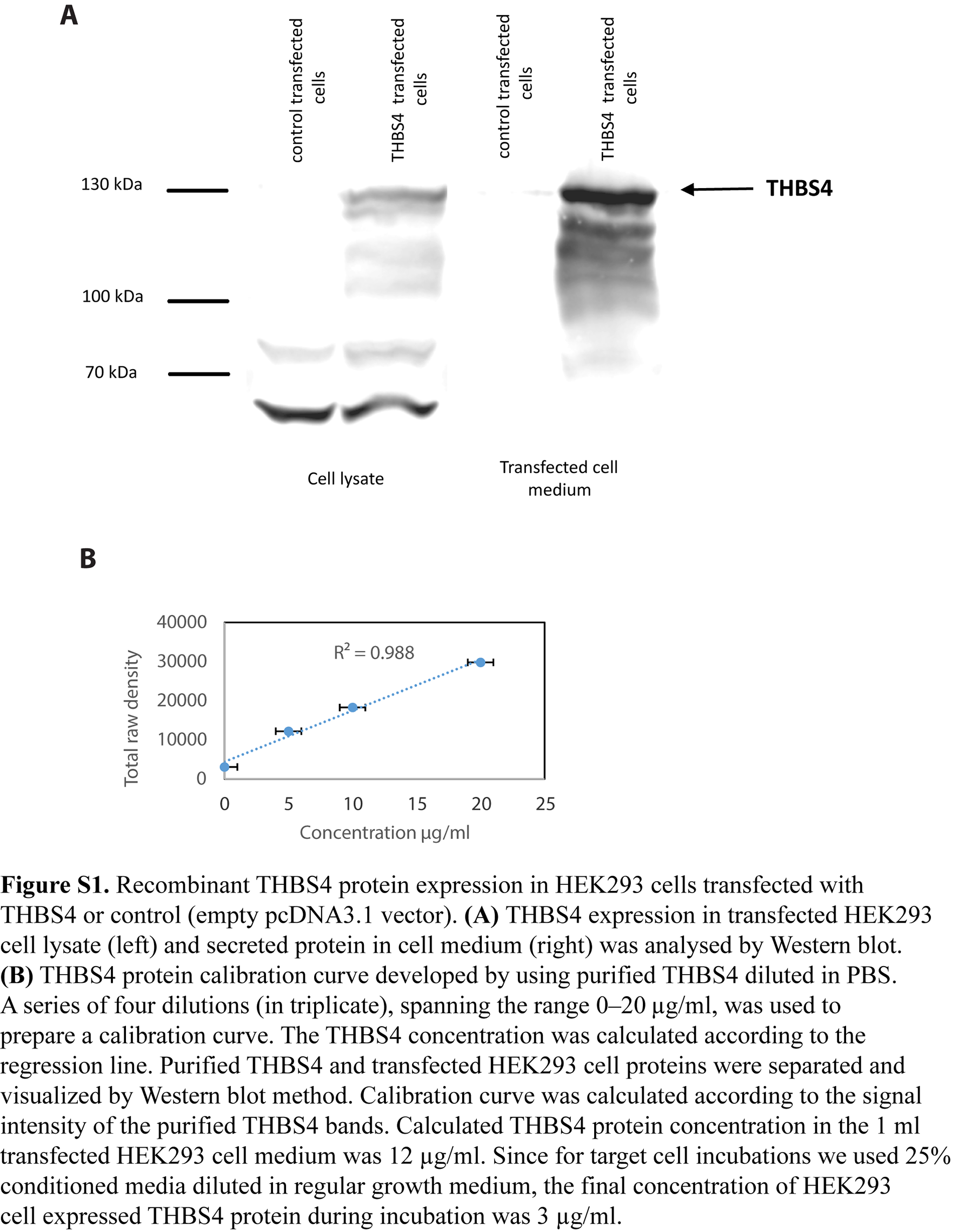

Supplement: Supplementary file 1 [file Image_1.TIF]

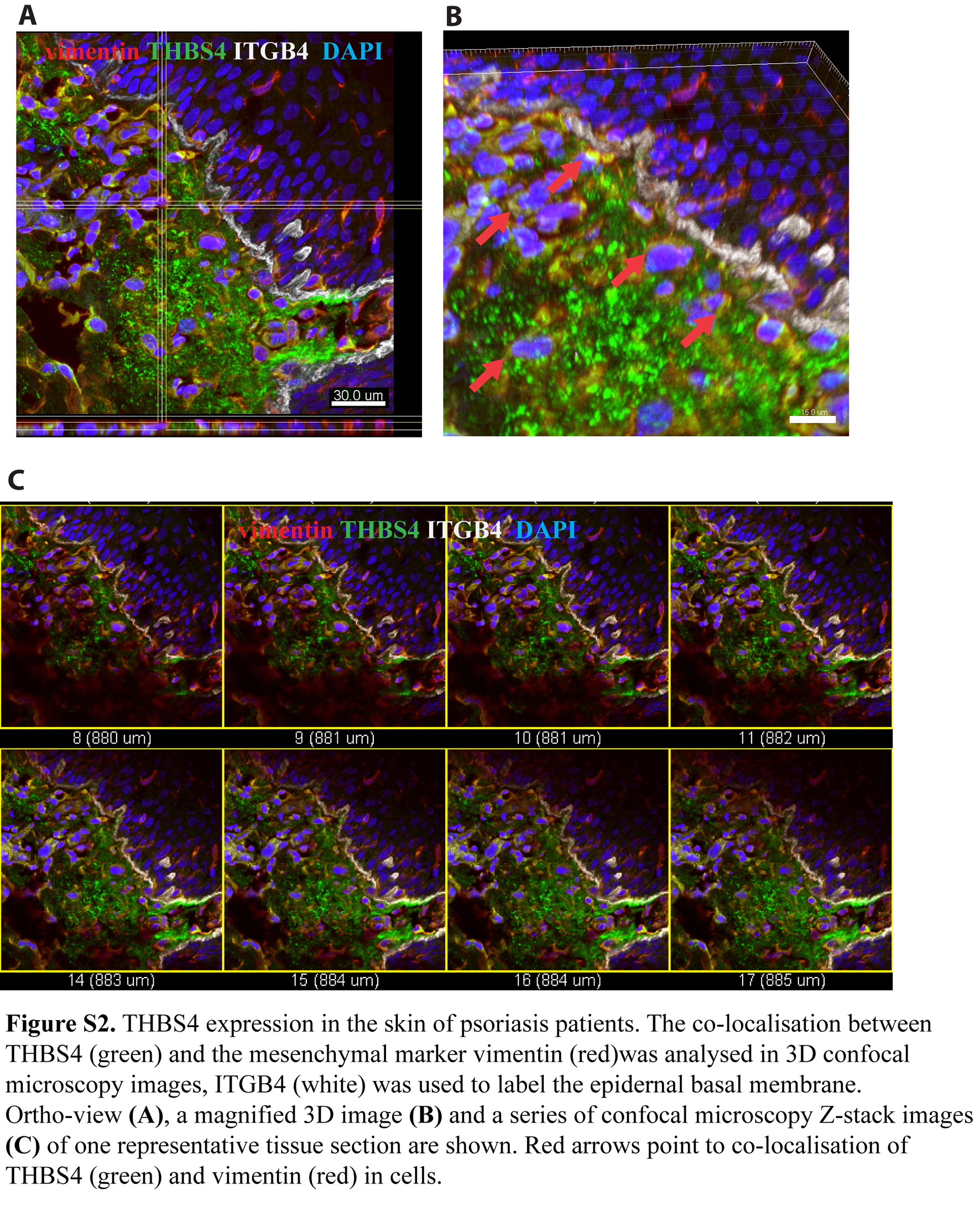

Supplement: Supplementary file 2 [file Image_2.TIF]

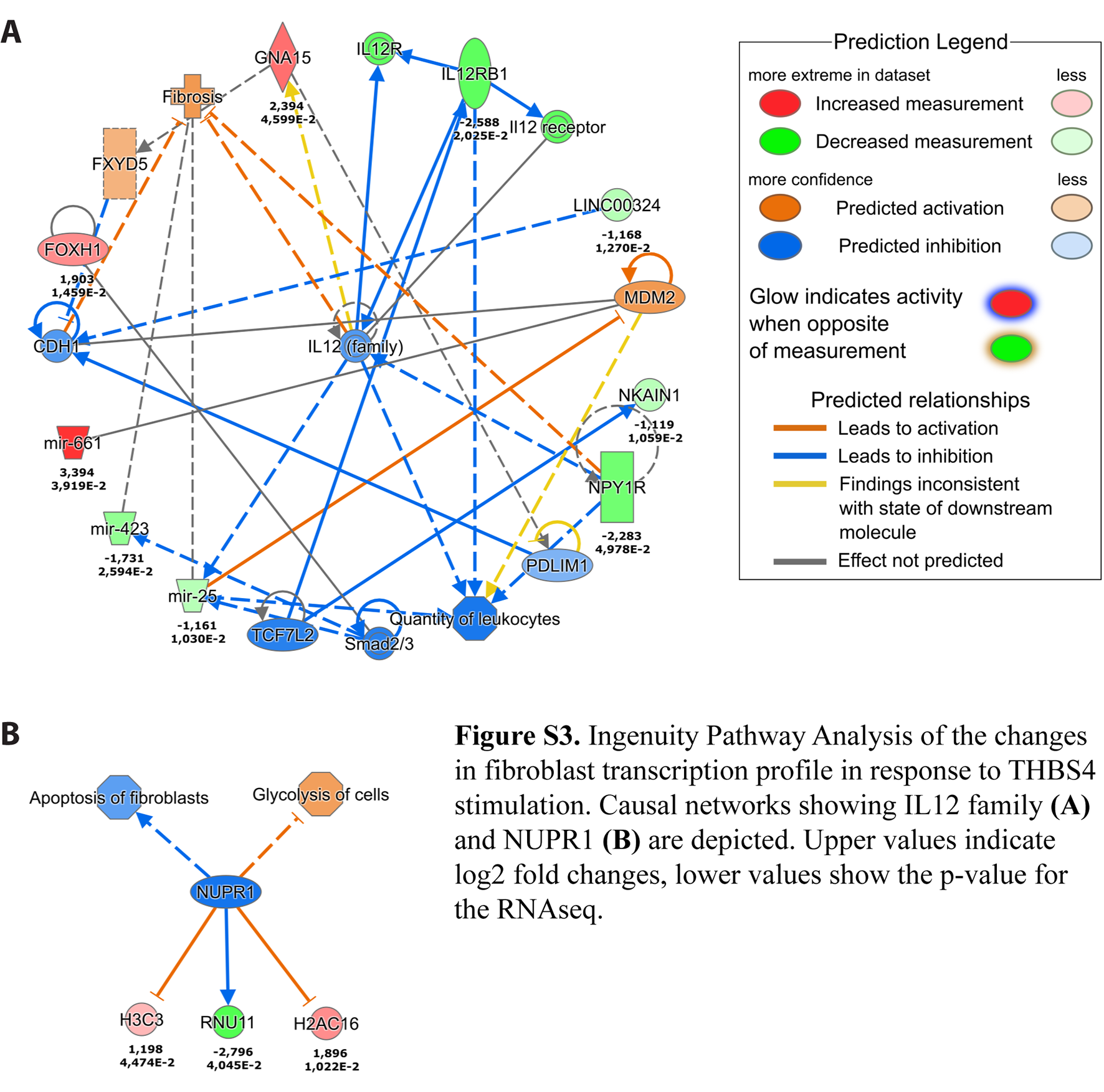

Supplement: Supplementary file 3 [file Image_3.TIF]

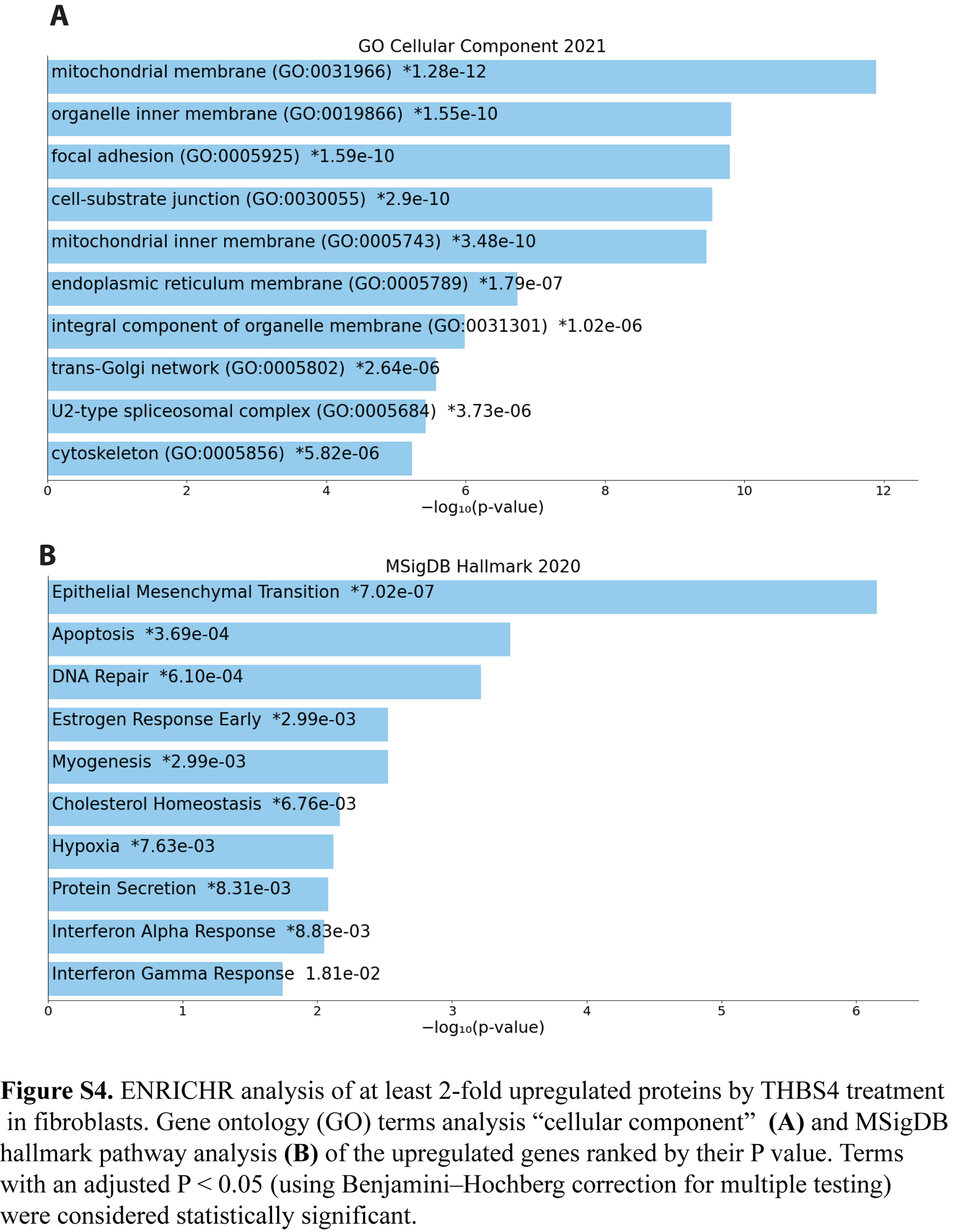

Supplement: Supplementary file 4 [file Image_4.TIF]

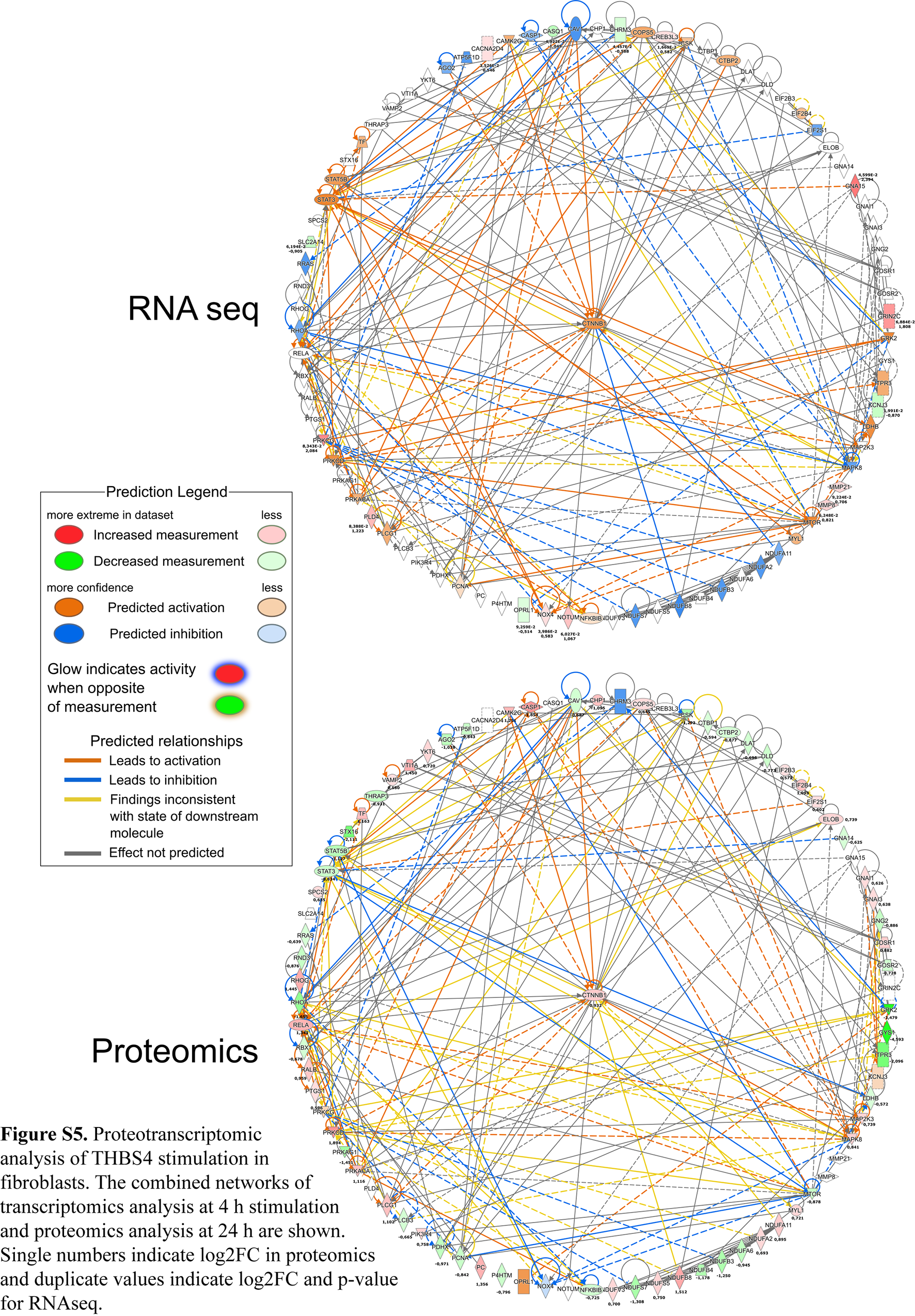

Supplement: Supplementary file 5 [file Image_5.TIF]

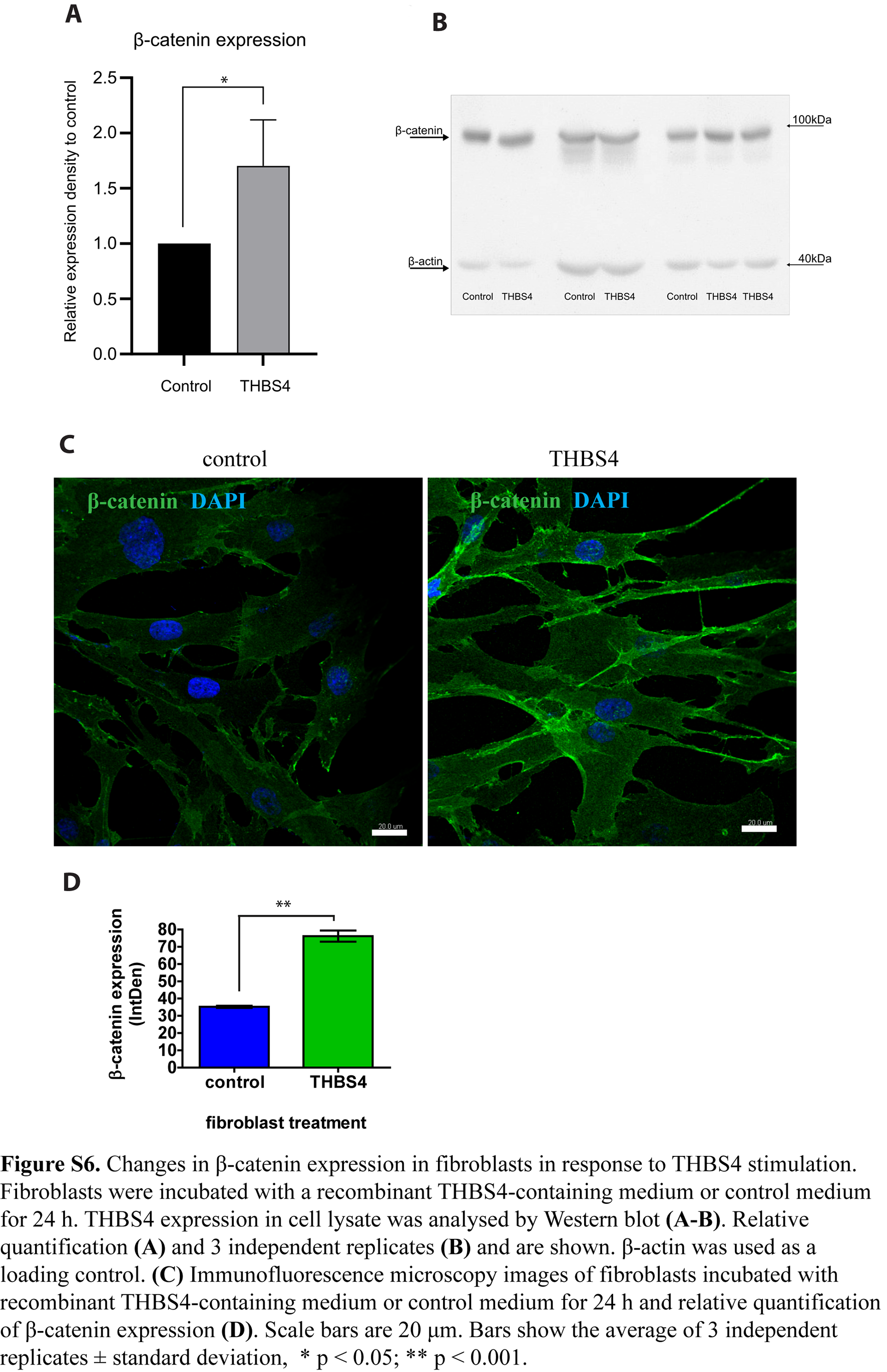

Supplement: Supplementary file 6 [file Image_6.TIF]

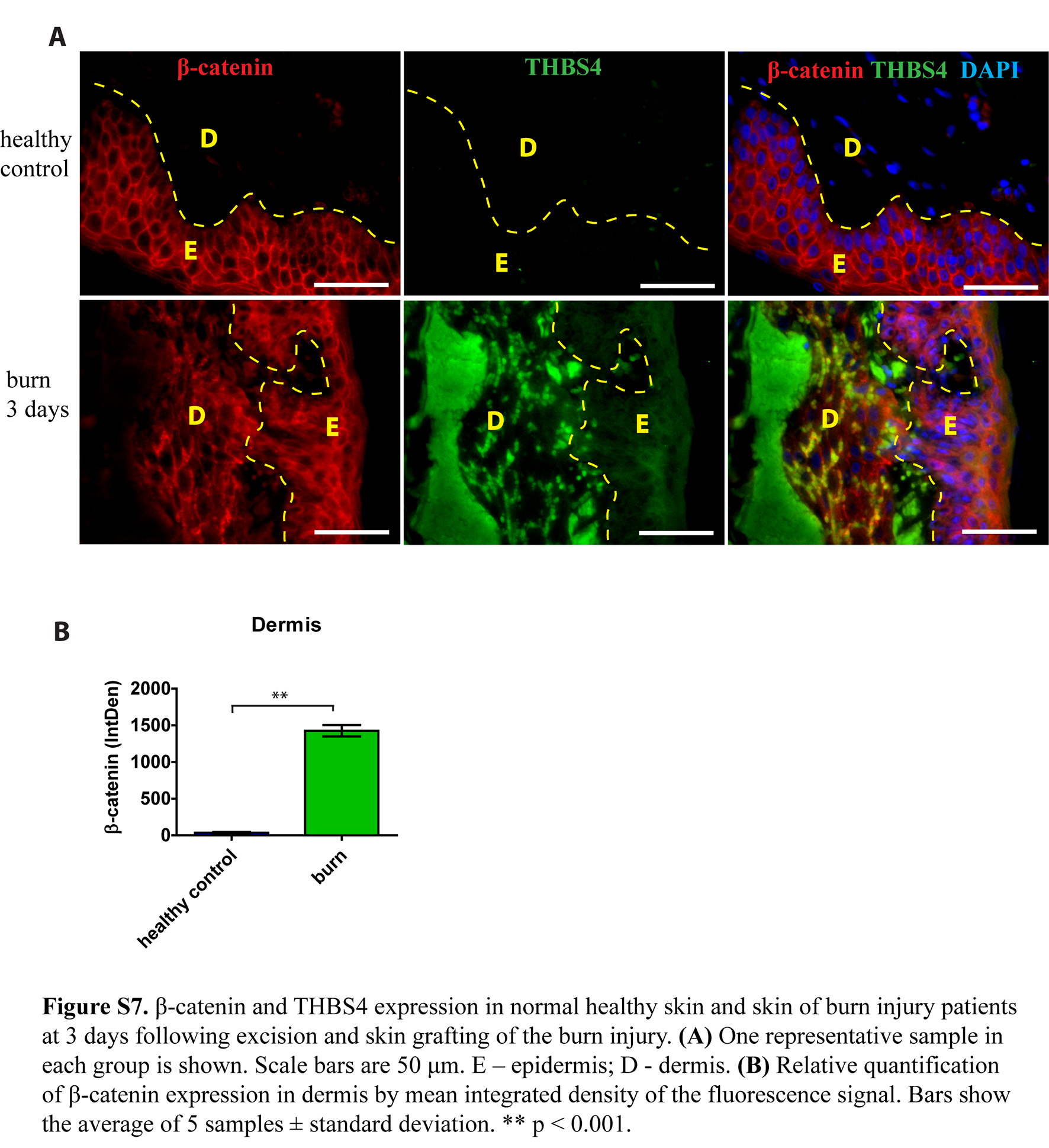

Supplement: Supplementary file 7 [file Image_7.TIF]

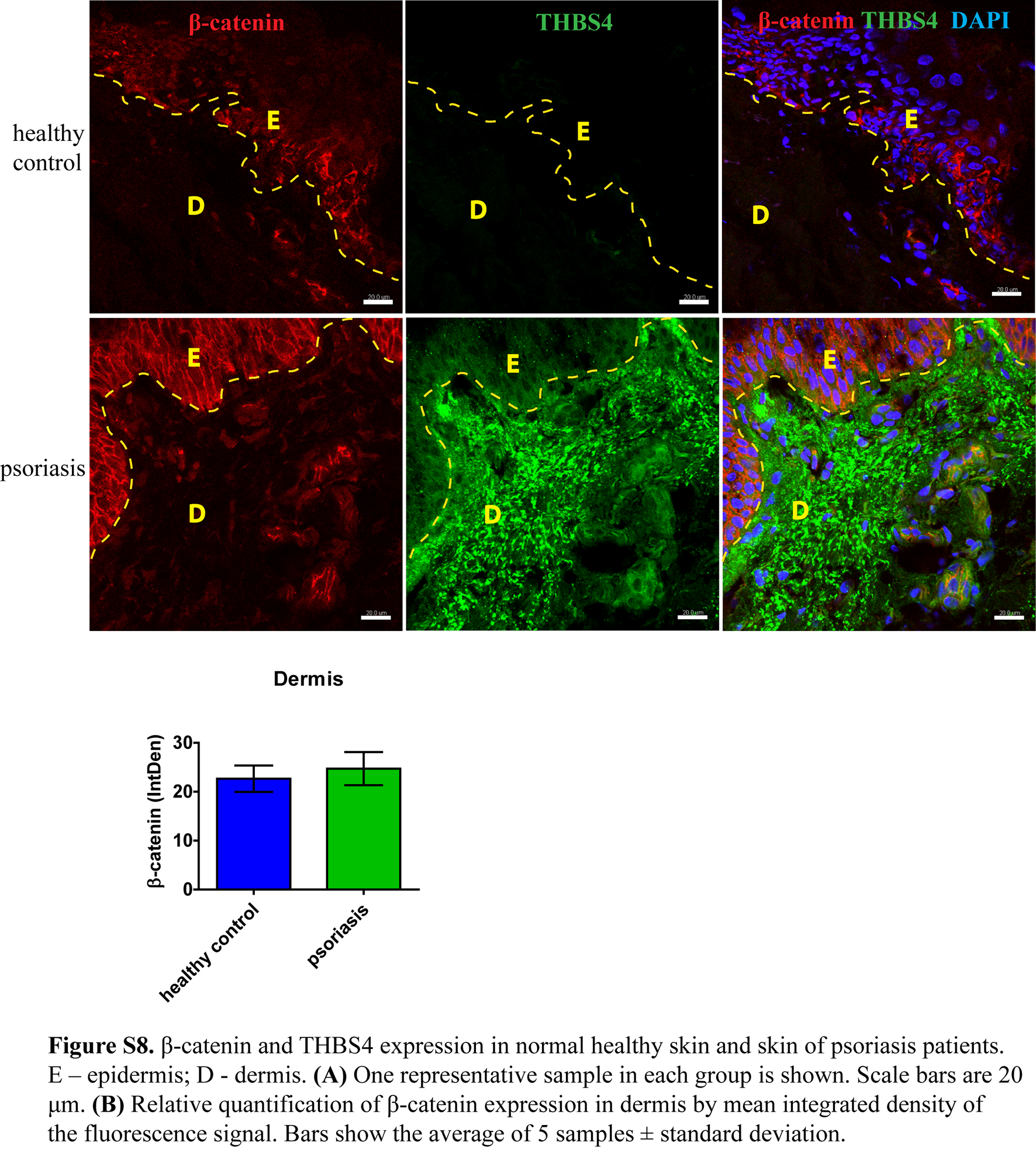

Supplement: Supplementary file 8 [file Image_8.TIF]
