# Supplementary material for: Thrombospondin-4 Is a Soluble Dermal Inflammatory Signal That Selectively Promotes Fibroblast Migration and Keratinocyte Proliferation for Skin Regeneration and Wound Healing
Source: Front Cell Dev Biol. 2021 Sep 23;9:745637. doi: 10.3389/fcell.2021.745637 (PMC8495264; doi:10.3389/fcell.2021.745637)
Supplement: Supplementary file 9 [file Table_1.DOCX]

**Supplementary Table S1.** Antibodies used in immunofluorescence analysis.

| **Antibody** | **Host, cat no** | **Dilution** | **Source** |
| --- | --- | --- | --- |
| Alexa Flour 488 Donkey anti-Goat | A11055 | 1:1000 | Thermo Fisher Scientific (Eugene, OR, USA) |
| Alexa Flour 488 Donkey anti-Rabbit | A21207 | 1:1000 | Thermo Fisher Scientific (Eugene, OR, USA) |
| Alexa Flour 568 Donkey anti-Rabbit | A10042 | 1:1000 | Thermo Fisher Scientific (Eugene, OR, USA) |
| Alexa Flour 647 Donkey anti-Mouse | A31571 | 1:1000 | Thermo Fisher Scientific (Eugene, OR, USA) |
| Integrin beta 4 | Mouse, FAB4060R-100UG | 1:200 | R&D Systems (Minneapolis, MN, USA) |
| Ki67 | Rat, 14-5698 | 1:200 | eBioscience (San Diego, CA, USA) |
| THBS4 | Goat, AF2390 | 1:100 | R&D Systems (Minneapolis, MN, USA) |
| Vimentin | Rabbit, ab92547 | 1:250 | Abcam (Cambridge, UK) |
