# Supplementary material for: Thrombospondin-4 Is a Soluble Dermal Inflammatory Signal That Selectively Promotes Fibroblast Migration and Keratinocyte Proliferation for Skin Regeneration and Wound Healing
Source: Front Cell Dev Biol. 2021 Sep 23;9:745637. doi: 10.3389/fcell.2021.745637 (PMC8495264; doi:10.3389/fcell.2021.745637)
Supplement: Supplementary file 10 [file Table_2.DOCX]

**Supplementary Table S2.** Proteomics analysis of fibroblasts stimulated with recombinant THBS4 for 24 h. MSigDB Hallmark 2020 database (Liberzon et al., 2015) was used for pathway analysis for proteins with at least 2-fold upregulation in response to THBS4 treatment.

| **Term** | **Overlap** | **P-value** | **Odds Ratio** | **Combined Score** | **Genes** |
| --- | --- | --- | --- | --- | --- |
| Epithelial Mesenchymal Transition | 21/200 | 7.017E-07 | 3.89 | 55.09 | JUN; SERPINE2; TPM4; IGFBP4; FUCA1; ELN; TPM1; MMP3; BGN; HTRA1; PLAUR; TNFRSF11B; DKK1; FBLN2; SGCD; CALD1; ABI3BP; COL6A2; CDH11; SLIT3; CCN2 |
| Apoptosis | 14/161 | 0.0004 | 3.12 | 24.69 | APP; BNIP3L; JUN; CDKN1A; HMGB2; BGN; RELA; EBP; PAK1; CASP8; CASP4; CASP1; TXNIP; IGFBP6 |
| DNA Repair | 13/150 | 0.0006 | 3.11 | 23.01 | SURF1; NCBP2; RFC2; RNMT; ALYREF; DCTN4; SMAD5; POLD3; BOLA2; ELOA; TMED2; NELFB; EIF1B |
| Estrogen Response Early | 14/200 | 0.0030 | 2.46 | 14.32 | IGFBP4; KRT8; KRT13; ABAT; NADSYN1; SEC14L2; SLC9A3R1; STC2; CCN5; RRP12; DHCR7; GLA; PTGES; FDFT1 |
| Myogenesis | 14/200 | 0.0030 | 2.46 | 14.32 | APP; WWTR1; CDKN1A; ACTN2; MYL6B; AEBP1; ENO3; SIRT2; SGCD; PC; STC2; COL6A2; ITGA7; MYH11 |
| Cholesterol Homeostasis | 7/74 | 0.0068 | 3.40 | 16.99 | EBP; ATXN2; MVK; PLAUR; DHCR7; ANTXR2; FDFT1 |
| Hypoxia | 13/200 | 0.0076 | 2.27 | 11.08 | BNIP3L; JUN; CDKN1A; BGN; PLAUR; IRS2; ENO3; NDST1; FAM162A; STC2; CCN5; CCN2; VHL |

**Reference**

Liberzon, A., Birger, C., Thorvaldsdottir, H., Ghandi, M., Mesirov, J.P., and Tamayo, P. (2015). The Molecular Signatures Database (MSigDB) hallmark gene set collection. *Cell Syst* 1**,** 417-425.
