# Supplementary material for: Thrombospondin-4 Is a Soluble Dermal Inflammatory Signal That Selectively Promotes Fibroblast Migration and Keratinocyte Proliferation for Skin Regeneration and Wound Healing
Source: Front Cell Dev Biol. 2021 Sep 23;9:745637. doi: 10.3389/fcell.2021.745637 (PMC8495264; doi:10.3389/fcell.2021.745637)
Supplement: Supplementary file 11 [file Table_3.DOCX]

**Supplementary Methods**

**Analysis of THBS4 expression in transfected HEK293 cells by Western blot**

HEK293 cells transfected with THBS4 plasmid or with an empty control plasmid backbone were lysed in a NP40 buffer containing protease inhibitors (Halt Protease Inhibitor Cocktail, Thermo Fisher Scientific, Rockford, IL, USA). After transfection, cells were incubated for 48 hours and conditioned medium was collected. For secreted protein analysis, conditioned media from both control- and THBS4-transfected cells were collected and precipitated with ice-cold acetone. After centrifugation at +4°C, the supernatant was discarded, and the protein pellet dissolved in MQ water. The samples with Laemmli buffer were heated at +95°C for 3 min before loading onto an SDS–PAGE gel. Separated proteins were transferred to PVDF membranes by electroblotting. THBS4 protein was detected by the primary antibody (Cat. AF2390, R&D Systems, Minneapolis, MN, USA) and the secondary reagent (Cat. E0466, Rabbit Anti-Goat Biotin and Streptavidin-AP, Dako, Glostrup, Denmark).

**Analysis of β-catenin expression in fibroblasts by Western blot**

Fibroblasts were cultured on 100mm cell culture dishes (Falcon Corning, Corning, NY, USA) until approximately 70% confluent and stimulated with recombinant THBS4-containing medium for 24h. Cells were lysed in an NP40 buffer containing proteinase inhibitors (Halt Protease Inhibitor Cocktail, Thermo Fisher Scientific, Rockford, IL, USA). The samples with Laemmli buffer were heated at +95°C for 3 min before loading onto an SDS–PAGE gel. Separated proteins were transferred to PVDF membranes by electroblotting. β-catenin expression was detected by the β-catenin primary antibody (Cat. #9581, Cell Signaling Technology, Danvers, MA, USA) and the secondary antibody (Cat. A16023, Donkey Anti-Rabbit Secondary Antibody, HRP conjugate, ThermoFisher, USA). A mouse monoclonal antibody (Cat. ab6276, Abcam, Cambridge, UK) was used for detection of β-actin as a sample loading control.

Densitometric quantification of Western blots was carried out using the FIJI processing package of ImageJ2 (Schindelin et al., 2012). Briefly, lanes were marked on 8-bit grayscale background-subtracted images using the ImageJ2 Gel analyzer-functions. Plotted densitometric peaks for beta-catenin and beta-actin were background-corrected and quantified as pixel areas. The area of each beta-catenin peak was first normalized to its corresponding beta-actin peak. Then each THBS4-treated sample's quantified beta-actin-normalized beta-catenin expression value was compared to that of its respective experiment control sample's value. Values from three independent experiments on a single gel were analyzed.

**Proteomics analysis**

Protein pellets were suspended in 25 µL of 7 M urea, 2 M thiourea, 100 mM ammonium bicarbonate (ABC), 2 mM methylamine solution, followed by disulphide reduction and cysteine alkylation with 5 mM dithiothreitol and 10 mM chloroacetamide for 60 min each at room temperature. Proteins were pre-digested with 1:50 (enzyme to protein) Lys-C protease (Thermo Fisher Scientific, Rockford, IL, USA) for 4 h, diluted 5 times with 100 mM ABC and further digested with trypsin overnight at room temperature. Peptides were desalted with in-house made C18 StageTips (Rappsilber et al., 2007) and reconstituted in 0.5% trifluoroacetic acid. 2 µg of the peptides were injected to an Ultimate 3000 RSLCnano system (Dionex, Sunnyvale, CA, USA) using a C18 cartridge trap-column in a backflush configuration and an in-house packed (3 µm C18 particles, Dr Maisch, Ammerbuch, Germany) analytical 50 cm x 75 µm emitter-column (New Objective, Woburn, MA, USA). Peptides were separated at 200 nL/min with a 5-40% B 240 min gradient. Buffer B was 80% acetonitrile + 0.1% formic acid and buffer A: 0.1% formic acid. Eluted peptides were sprayed to a quadrupole-orbitrap Q Exactive Plus (Thermo Fisher Scientific, Waltham, MA, USA) tandem mass spectrometer (MS) using a nano-electrospray source and a spray voltage of 2.5 kV (liquid junction connection). The MS instrument was operated with a top-10 data-dependent acquisition strategy. One 350-1400 m/z MS scan (at a resolution setting of 70,000 at 200 m/z) was followed by a MS/MS (R=17,500 at 200 m/z) of the 10 most intense ions using higher-energy collisional dissociation fragmentation (normalized collision energies of 26 peptides). The MS and MS/MS ion target and injection time values were 3x 106 (50 ms) and 5x 104 (50 ms), respectively. The dynamic exclusion time was limited to 45 s and 60. Only charge states +2 to +6 were subjected to MS/MS. For the MaxQuant (Cox and Mann, 2008) identification and quantification of raw MS data, UniProt ([www.uniprot.org](http://www.uniprot.org), release [2020_06](https://www.uniprot.org/news/2020/12/02/release)) human reference proteome database was used. Tryptic digestion rule (cleavages after lysine and arginine without proline restriction) was used for *in silico* digestion of the database. Only identifications with at least 1 peptide ≥ 7 amino acids long (with up to 2 missed cleavages) were accepted. Peptide-spectrum match and protein false discovery rate (FDR) were kept below 1% using a target-decoy approach. All other parameters were default.

**References**

Cox, J., and Mann, M. (2008). MaxQuant enables high peptide identification rates, individualized p.p.b.-range mass accuracies and proteome-wide protein quantification. *Nature Biotechnology* 26**,** 1367-1372.

Rappsilber, J., Mann, M., and Ishihama, Y. (2007). Protocol for micro-purification, enrichment, pre-fractionation and storage of peptides for proteomics using StageTips. *Nature Protocols* 2**,** 1896-1906.

Schindelin, J., Arganda-Carreras, I., Frise, E., Kaynig, V., Longair, M., Pietzsch, T., Preibisch, S., Rueden, C., Saalfeld, S., Schmid, B., Tinevez, J.Y., White, D.J., Hartenstein, V., Eliceiri, K., Tomancak, P., and Cardona, A. (2012). Fiji: an open-source platform for biological-image analysis. *Nat Methods* 9**,** 676-682.
